# Comparison of Performance Criteria of Vehicle Brake Pad Biopolymers Derived from Renewable Biomass

**DOI:** 10.3390/polym18080950

**Published:** 2026-04-13

**Authors:** Hicri Yavuz

**Affiliations:** Department of Engine Vehicles and Transportation Technology, Afyon Vocational School, Afyon Kocatepe University, Afyonkarahisar 03200, Türkiye; hicriyavuz@aku.edu.tr; Tel.: +90-505-510-64-66

**Keywords:** polymers, brake pad, coefficient of friction, wear, composites

## Abstract

In this article, four grades of brake pads reinforced with loofah fiber were developed, serving as examples of renewable biomass-based products currently receiving significant attention. In addition to the performance characteristics of the developed brake pads, such as friction coefficient and wear rate, macroscopic and microscopic surface analyses of the worn surfaces were performed. Furthermore, unlike other studies in this field, the effect of biopolymer brake pads on the brake disk surface was also investigated. The average friction coefficients of the brake pads are 0.32, 0.28, 0.28, and 0.27, respectively, and the developed brake pads are within industrial limits of 0.20–0.70. The wear rates were also found to be within industrial limits, with values of 2.6 × 10^−8^ cm^3^/Nm, 1.47 × 10^−8^ cm^3^/Nm, 1.28 × 10^−8^ cm^3^/Nm, and 1.05 × 10^−8^ cm^3^/Nm, respectively. It was determined that the differences in brake disk roughness were also at the desired level for all samples. The desired results in brake friction materials were confirmed by macroscopic and microscopic surface examinations, as well as other properties of the developed samples.

## 1. Introduction

Today, due to climate change, environmental events, and concerns about production and consumption, industrial firms and researchers are making intensive efforts across various fields to advance green environments, sustainability, and renewability. Undoubtedly, the most important area in these efforts is the development of biopolymers from biomass-based products. Automotive engineering is one of the leading industrial fields in this area of research. While the development of biopolymer materials for various components in the automotive sector is increasing, research on brake pads, a part of the braking system, has become an inevitable necessity.

The braking system is a crucial component of automotive safety. The braking mechanism’s performance at its desired level is closely related to the quality and durability of the brake pads. These pads are responsible for reducing or stopping the wheels [1]. The braking system, which performs this task, converts the kinetic energy of the wheels into heat through friction. As a result of this conversion, the vehicle should stop or slow down. Among the most important parts of the braking system are the brake disk, brake drum, and brake pads, which work together and play a decisive role in determining braking performance [2]. Disk brake systems, an important part of braking systems, consist of a rotor (or brake disk) to which a caliper applies pressure, and a brake friction material, also known as a brake pad [3]. To achieve a long service life in braking systems, efforts are made to develop brake materials with high friction coefficients. Due to environmental concerns, agricultural products and their waste, as well as industrial waste, are being used as alternatives to materials such as asbestos [4]. For these modern applications, new brake pad friction products with desired properties are developed. Developing brake friction materials that meet the desired property requirements remains a complex, unresolved issue [5].

Developing brake pads that meet the desired criteria requires balancing acceptable performance standards with cost-effective and environmentally friendly applications [6]. These initiatives aim to reduce both health and environmental risks by replacing conventional materials, such as asbestos or copper, with alternatives like walnut shells or other so-called eco-friendly options [7]. Along with this need, interest in plant-based fibers has undoubtedly increased worldwide due to factors such as environmental sustainability, reduced carbon footprints, and a shift towards alternative energy sources like electricity [8,9,10]. The increasing interest is mainly due to the impact of unsustainable failures. Because of these effects, the use of biofibers in various composite applications is attracting significant attention [11].

A review of the literature reveals that brake pads have been developed using a wide variety of materials [12,13,14]. Brake friction materials are primarily developed by combining components, including reinforcing agents, fillers, binders, lubricants, and abrasives [15]. Studies in this area reveal a vast potential for the use of natural fibers in the brake pad industry. This diversification in the sector leads to the use of agricultural waste and natural products, while also raising environmental and health concerns. Asbestos-free organic brake pads, also known as NAO or organic brake friction pads, are produced by using various natural fibers combined with resin [7]. These newly developed products have been found to perform almost as well as conventionally manufactured brake pads [1]. These natural fiber-reinforced products are considered promising materials due to their desirable mechanical properties and eco-friendliness [16]. The reasons why these materials have become a focal point include their availability in nature, their mechanical properties, their light weight, their low cost, and their easy biodegradability in the environment [17]. These materials include pampas grass stalks, Blue Cupressus Arizona Cone, hemp fiber, coconut fiber, wood dust or flour, bark powder, or bamboo fiber. These materials are widely used as reinforcement materials because of their high specific strength, light weight, eco-friendliness, biodegradability, and low cost [1,18,19].

The main objective of this study is to systematically investigate and experimentally demonstrate the potential of loofah fiber, a material with a rigid fiber structure, as an alternative reinforcement element in brake pads. The primary reasons for choosing loofah fiber in this study are, firstly, that, according to information obtained from literature reviews, this type of fiber has not been previously studied in any brake pad formulation in varying proportions along with other materials used in the study. Secondly, loofah fiber offers significant advantages, including sustainability, renewability, and environmental friendliness. Thirdly, as a biomass-based product, it is a potential raw material that can help reduce production costs. The study aims to determine the effects of loofah fiber, considered as a reinforcing material, on brake pad performance.

This article describes the development of four variants of loofah fiber-reinforced polymer brake pad compositions via hot molding. In addition to the performance of these developed materials, unlike other studies, the effect of the brake pads on the brake disk with which they interact was also investigated. Micrographs of the worn surfaces were examined using scanning electron microscopy (SEM, LEO brand, 1430 VP Model, Carl Zeiss AG, Jena, Germany). Macroscopic images of the worn surfaces were also examined using a USB microscope (Generic brand, 1000X Model, Shenzhen, China) to provide insights into larger contact surfaces.

The results showed that, except for the BPLY8 sample, the other three samples provided stable friction coefficients and wear rates at levels specified in the TS555 [20] standards for vehicles and could also contribute to the formation of a desired brake friction material.

## 2. Materials and Methods

### 2.1. Materials

**Figure 1:**
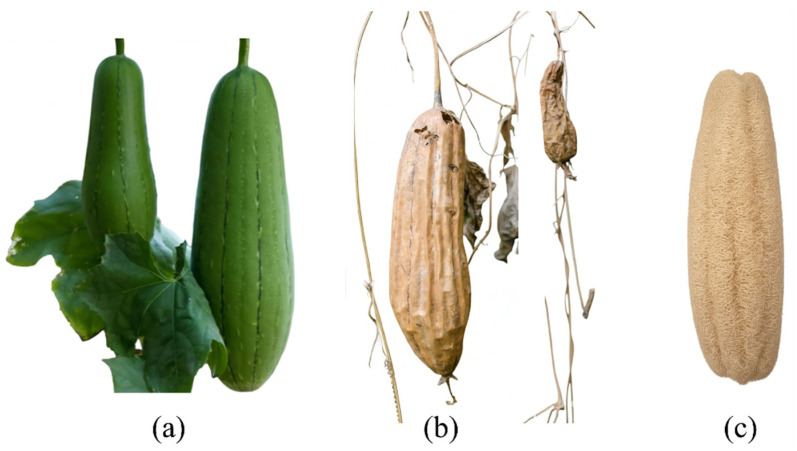
Processes for obtaining the final product from loofah gourd, (**a**) Green loofah; (**b**) Loofah drying on the branch; (**c**) Loofah with peeled off.

The materials used in the sample, including loofah fiber, were used without any chemical or physical processing. The stages of loofah fiber production, from cultivation in nature to the final product, are shown in Figure 1. Figure 1a shows fresh green loofahs growing in nature and hanging on the branch, Figure 1b shows loofahs that have started to dry, turning from green to brownish in nature, and Figure 1c shows the renewable biomass-based fiber after its outer shell has been peeled off and it has been made ready for use. After the final product process, the natural fibers were cut into small pieces with a maximum shear gap of 3 mm by shearing, without any chemical processing, and were made ready for use.

### 2.2. Sample Preparation

The mixing ratios of the materials forming the biopolymer brake pad composites are shown in Table 1.

The biopolymer-based samples are abbreviated as BPLY. The numbers at the end of this abbreviation indicate the percentage by weight of the amount of loofah fiber present in the sample.

Previous literature [21,22,23,24] was considered in the methods used to develop brake pads. For production, the materials were weighed on a balance with a sensitivity of 1 mg in the ratios specified in Table 1. To ensure homogeneous mixing of the weighed materials, a mixing process was carried out for 15 min at 60 rpm in a kinetic three-dimensional mixer. The mixed materials were then weighed again on a precision balance, each weighing 10 g. The weighed mixture was discharged into each chamber of a six-chambered hot mold. During production, the mold pressure was set to 40 MPa, the temperature to 160 °C, and the molding time to 15 min per sample.

### 2.3. Research Methods

Brake pads are expected to operate stably under varying temperature and load conditions. To evaluate this performance compatibility, controlled tests are conducted within the limits defined by the standards. The variation in the friction coefficient and wear rate over a sliding distance of 10,800 m was tested on a brake pad testing device at a linear speed of 6 m/s and a load of 1 MPa, within the limits specified by the relevant TS555 [20] standard. The creep coefficient and the equation used to calculate it are given in Equation (1), and the equation used to calculate the wear rate is given in Equation (2). The brake pad densities required to achieve the specified wear rate were calculated using Archimedes’ principle.

(1) $$\mu =\frac{f}{F}$$

where *μ*: Coefficient of friction of brake pad samples (*μ*), *f*: Friction force read on the brake test device (N), *F*: Load applied to brake pad samples (N)

(2) $$W_{a}=\frac{∆G}{S\times M\times d}$$

where *W_a_*: Wear rate (cm^3^/Nm), Δ*G*: Mass loss before and after the test (g), *S*: Brake pad sliding distance (m), *M*: Applied load value (N), and *d*: Density of brake pads (g/cm^3^).

Hardness testing of brake friction materials was performed to determine the material’s resistance to deformation and to permanent indentation caused by chipped pieces [25]. Hardness tests were performed according to ASTM D2240 [26]. For each series, the tests were conducted by averaging the Shore D hardness values measured at five different points on three samples.

The effect of brake pad samples on the disk they work with is important for interpreting the service life and interaction of these parts. Unlike most studies in the literature, this study determined the average roughness (Ra) and maximum roughness (Rz) values produced by brake pad samples on the brake disk over a sliding distance of 21,600 m. Before each measurement, the disk surface was sanded with 320-grit sandpaper at the beginning of the experiment. Subsequently, measurements were taken at 3 points, equally spaced on the disk, using a two-dimensional roughness measuring device with a measurement interval of 40 µm and a measurement length of 2.5 mm. The differences between the first and last measurements were determined by averaging the measured values.

To visualize micrographs of worn surfaces, microscopic analyses are performed on brake pad samples using a Scanning Electron Microscope (SEM) [18]. In this study, SEM analyses were performed on the worn brake pad surfaces from each sample series. Based on the resulting images, SEM analyses were performed. In addition, macroscopic analyses of the samples’ worn surfaces were performed using a USB microscope to observe deformations that may occur over large areas of the sample surface.

## 3. Results and Discussions

### 3.1. Study on Coefficient of Friction

**Figure 2:**
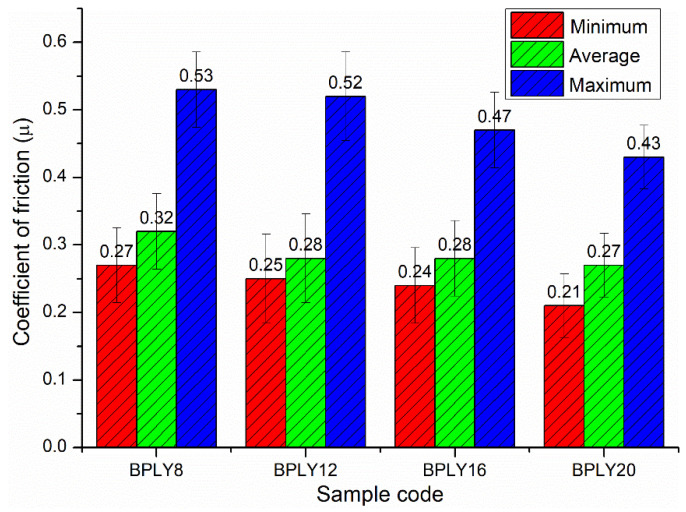
Friction coefficients of brake pad biopolymers.

The minimum, average, and maximum values of the friction coefficient for the brake pad samples are shown in Figure 2. The minimum friction coefficient of the brake pads was obtained as 0.21, and friction coefficient values above this value were determined in the other samples. The average friction coefficient was 0.27 in the BPLY20 sample. According to the results, the average friction coefficient decreased with increasing fiber ratio in the sample. However, it remained within the desired friction coefficient values. According to the standards [20], the friction coefficient should be between 0.20 and 0.70 for safe, effective braking. When the findings in Figure 2 are examined, it is seen that the friction coefficient values obtained for all samples remained within the specified range. This shows that the developed luffa fiber-reinforced brake pads exhibit acceptable, safe friction performance even under variable-temperature conditions.

Due to adhesive wear during the initial break-in period between the disk and the brake pad, a higher adhesion force is generated between the brake friction material (pad) and the metal disk [18]. This adjustment mechanism gradually stabilizes the uneven distribution of contact pressure on the friction block surface between the disk and the brake pad as the sliding distance increases, thereby stabilizing the surface along the direction of disk rotation, i.e., the direction of friction [27]. In this second regime, the friction coefficient values become more consistent and, consequently, more stable [18].

**Figure 3:**
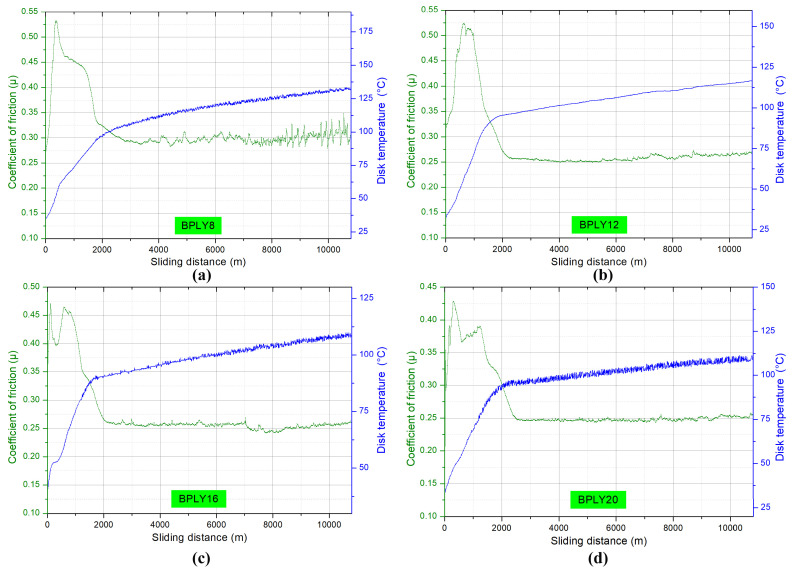
Friction coefficient and temperatures of brake pad biopolymers, (**a**) BPLY8; (**b**) BPLY12; (**c**) BPLY16; (**d**) BPLY20.

Figure 3a shows the instantaneous coefficient of friction and temperature graphs as a function of sliding distance for BPLY8, Figure 3b BPLY12, Figure 3c BPLY16, and Figure 3d BPLY20 samples under dry sliding conditions. The graphs in Figure 3 confirm that the friction phenomena described above occur. In all brake pad samples, the coefficient of friction tended to increase up to approximately 1000 m of sliding distance due to a higher adhesion force. This increasing trend decreased to 2000 m of sliding distance due to increased temperature above 1000 m, resulting from friction between the disk and the pad.

Generally, during braking tests, the temperature at the brake pad-disk interface tends to increase with increasing slip distance [27]. In vehicles, the brake pad friction materials should maintain a stable braking force and therefore a stable coefficient of friction under varying conditions. Consequently, a stable coefficient of friction is a desirable outcome for drivers [28]. During the experiment, the standard deviation values for the samples were calculated as 0.05565, 0.06579, 0.05598, and 0.04703, respectively. Considering the lower-temperature increase trend from the 2000 m sliding distance onward and the standard deviation findings together, it can be stated that the friction coefficient exhibits a relatively more stable behavior over sliding distance. When the graphs are examined after a certain sliding distance, it is seen that the sample coded BPLY8 shows significant fluctuations from 8000 m onwards. In contrast, the other samples exhibit slightly lower friction coefficients than BPLY8 in the 5000–10,000 m range. The stable friction coefficient obtained in the developed samples indicates that the use of loofah fiber in brake pads can deliver the expected performance.

### 3.2. Study on Wear Rate and Density

**Figure 4:**
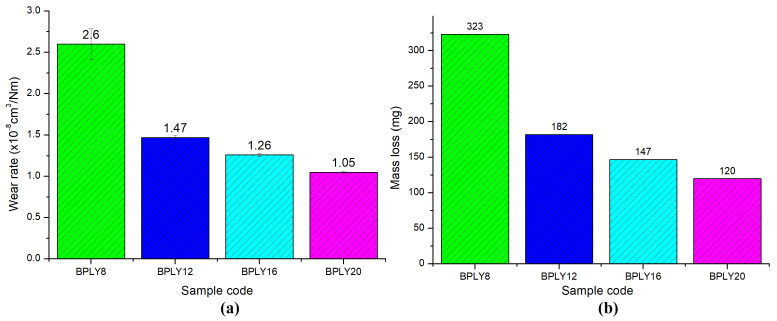
Wear rates of brake pad biopolymers, (**a**) specific wear; (**b**) mass wear.

In the density test conducted to calculate the wear rates, the densities of the samples containing 8%, 12%, 16%, and 20% loofah fiber were measured as 2.234 g/cm^3^, 2.160 g/cm^3^, 2.088 g/cm^3^, and 2.029 g/cm^3^, respectively. According to the measured results, as the fiber content in the brake pad samples increased, their density decreased. This situation will make them advantageous for reducing brake pad weight. Figure 4a shows the wear rate values of the brake pad samples in cm^3^/Nm, and Figure 4b shows the wear rate values in mass. In the bar graphs shown in Figure 4a,b, the green bar represents the data for sample BPLY8, the blue bar for sample BPLY12, the turquoise bar for sample BPLY16, and the purple bar for sample BPLY20.

In brake friction materials, there is a direct, proportional relationship between the material’s wear resistance and the increase in temperature during braking. That is, the higher the temperature, the lower the brake pads’ wear resistance. This is one of the important factors that leads to the use of multi-material components in the development of brake friction materials [29]. Maximum temperatures of 133 °C were obtained in sample BPLY8, 117 °C in sample BPLY12, 110 °C in sample BPLY16, and 111 °C in sample BPLY20. As shown in Figure 4, the wear rate values showed a decreasing trend from high to low, from the sample containing 8% fiber to the sample containing 20% fiber. This pattern of results can be attributed to the decrease in temperature values, as explained above.

Another factor influencing wear rate is the direct correlation between the coefficient of friction and the wear rate. As the coefficient of friction increases, the wear rate tends to increase as well [30]. When Figure 4a,b are examined together in Figure 2, as can be seen in Figure 2, the average friction coefficient shows a decreasing trend as the fiber content in the sample increases. In direct proportion to this, as shown in Figure 4, the friction coefficient decreases with increasing fiber content. As a result of the increased fiber content, the reduced wear residues and increased reinforcing properties also reduce wear rates. Theoretically, according to accepted standards in the brake pad industry (TS 555 [20]), the wear rate of brake pads is considered ideal to be between 0.5 × 10^−7^ cm^3^/Nm and 3.5 × 10^−7^ cm^3^/Nm. Reference values are crucial for ensuring safe, stable performance throughout the brake pad’s service life. In this study, the wear rates obtained from wear tests on developed luffa fiber-reinforced brake pad samples were examined. It was determined that the calculated wear rates for the developed samples remained within the standard reference range. This indicates that the developed brake pads meet industrial requirements for wear resistance and are safe to use.

### 3.3. Study on Hardnesses

**Figure 5:**
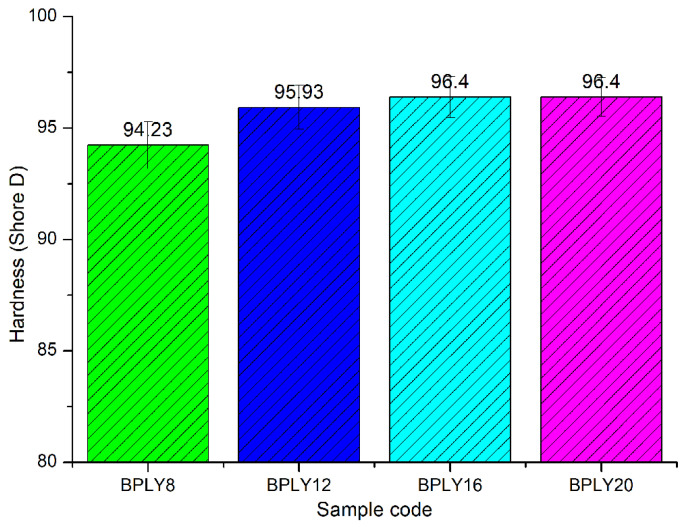
Hardness of brake pad biopolymers.

Figure 5 shows the Shore D hardness graph of the brake pad samples. In the bar graphs shown in Figure 5, the green bar represents data for sample BPLY8, the blue bar for sample BPLY12, the turquoise bar for sample BPLY16, and the purple bar for sample BPLY20. As shown in the graph, the hardness values showed a very slight upward trend as the fiber content in the sample increased. This slight difference in hardness values indicates that the formulation process during sample development was highly controlled and that the material properties remained relatively consistent [25]. In brake pads, sample hardness is generally considered an influential factor in increasing or decreasing the wear rate. Typically, as sample hardness increases, the sample’s wear rate decreases. This study has shown that increasing sample hardness, even partially, as fiber content increases, reduces the wear rate.

### 3.4. Study on Difference in Roughness

**Figure 6:**
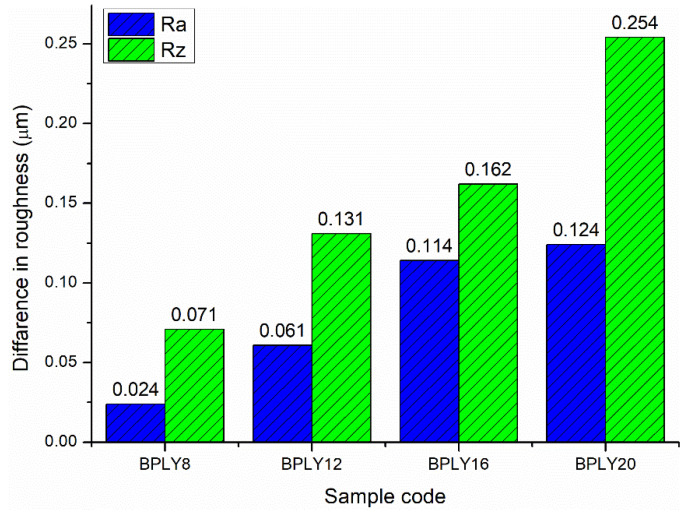
Differences in brake disk roughness caused by brake pad biopolymers.

**Figure 8:**
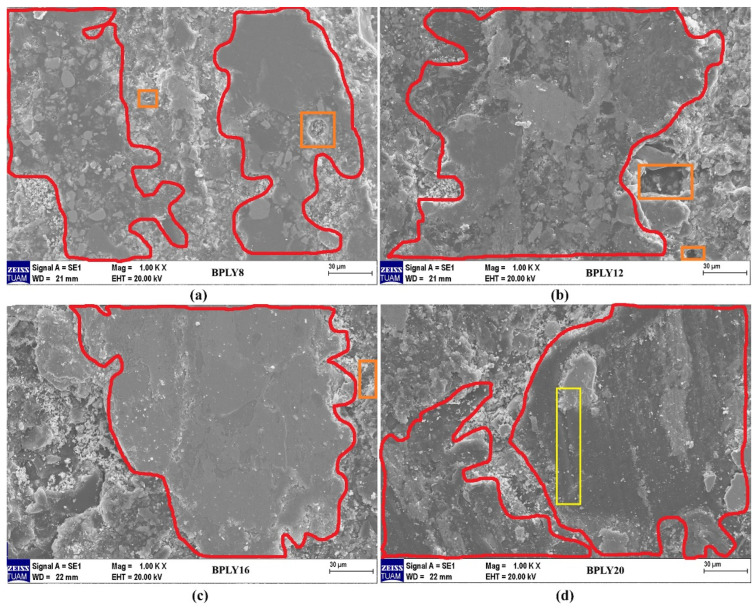
SEM images of brake pad biopolymers, (**a**) BPLY8; (**b**) BPLY12; (**c**) BPLY16; (**d**) BPLY20.

The long lifespan of brake disks, which work in conjunction with brake pads, is a desirable feature for users. Considering this aspect during brake pad development is a crucial point for both industrial companies and researchers [21,31]. Increased disk roughness in vehicles significantly impacts braking performance. On the positive side, a certain level of surface roughness, especially if there is no glazing on the brake disk surface, increases the coefficient of friction between the brake pad and the disk, naturally leading to improved braking efficiency. Shorter stopping distances can be achieved, particularly at low vehicle speeds. However, exceeding a certain threshold of roughness or accelerated disk wear can lead to increased brake noise and vibration, as well as shorter brake pad life. For optimal brake system design, the disk surface roughness should be within a specific range to balance wear, noise, and vibration from braking. Figure 6 shows the differences in Ra and Rz roughness values measured before and after the experiment on brake pads. disk roughness difference is a necessary condition that will arise in the brake pad-disk interaction during braking. This is because there is a friction-based relationship between the two surfaces, and as a natural consequence, roughness differences will occur. As the fiber content in the sample increased, the average and maximum roughness differences before and after the experiment also tended to increase. The hardness of the samples also affects this situation; as the sample hardness increased, the disk roughness differences also tended to increase. Differences in roughness on the disk surface can also be evaluated by analyzing abrasive particles at the friction interface. For sample BPLY20, a distinct abrasive wear trace is observed in the region shown within the yellow rectangle in Figure 8d. This wear trace directly contributes to the higher roughness difference in sample BPLY20 compared to other samples. The abrasive wear mechanism identified in this region contributes to the increase in roughness values along with the deterioration of the disk surface roughness. Compared to other studies in the literature [21,31], the disk roughness differences are at a reasonable level for loofah fiber reinforcement.

### 3.5. Study on the Macroscopic and Microscopic Structure of Worn Surfaces

**Figure 7:**
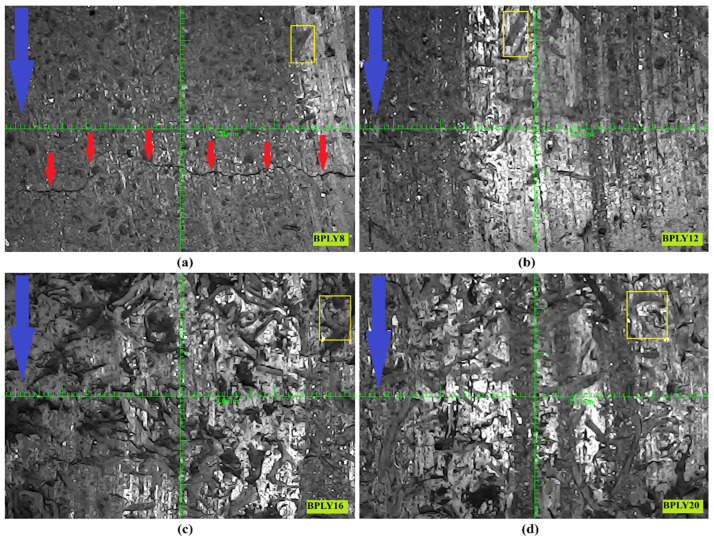
Macroscopic images of the worn surface of brake pad biopolymers, (**a**) BPLY8; (**b**) BPLY12; (**c**) BPLY16; (**d**) BPLY20.

Figure 7a shows the macroscopic image of the worn surfaces of BPLY8, Figure 7b shows BPLY12, Figure 7c shows BPLY16, and Figure 7d shows BPLY20. Considering the elongated, fibrous structure, indistinct aspect ratio, and irregular geometric patterns of the particles located in the regions marked within the yellow rectangle in the macroscopic images, it is assessed that these structures belong to pumpkin fibers distributed within the matrix phase. The fact that the particles exhibit a fibrous and elongated shape rather than a spherical or angular geometry strongly suggests that they reflect the morphological characteristics of pumpkin fibers present in the matrix. In these images, the yellow rectangles indicate fiber images within the matrix.

As shown in Figure 7a–d, the fiber images in the yellow rectangular area tend to increase on the surface as the fiber ratio in the sample increases. This proves that fibers are homogeneously distributed in brake pad samples. The blue arrow symbol in the upper left corner of all images in Figure 7 indicates the direction of rotation of the brake disk. The fiber ratio used as a reinforcing material in brake pads is important. This is because the reinforcing fibers ensure that the materials forming the brake pads remain together and integrated under mechanical loads during braking. In studies in this field, researchers are trying to determine the optimal fiber content. Observations made after the experiment revealed that in the BPLY8 sample, a macroscopic fracture occurred along the entire length of the brake pad in the direction of disk rotation, in the areas indicated by the red arrow in Figure 7a. These fracture patterns, which occur after braking due to mechanical loads, are caused by a reinforcement material ratio that is insufficient to hold the brake pad materials together. When macroscopic images were examined, it was determined that similar crack formation did not occur in any of the samples except the BPLY8 sample. Crack formation was observed in the BPLY8 specimen after the experiment. This strengthens the possibility that crack formation may be effective in the observation of a more unstable coefficient of friction in the BPLY8 specimen after a sliding distance of 8000 m in Figure 3a. Therefore, we concluded that an 8% loofah fiber content is not an optimal ratio for brake pads.

In brake pads, which are called polymer matrix composites, the microscopic structure of wear mechanisms, fiber deformations, wear-residue formation, fiber and matrix separations, cracks that may occur on the composite surface, and, most importantly, contact plateaus are of great importance [18,32]. After dynamometer testing of brake pads, the worn pad surfaces are analyzed using a scanning electron microscope (SEM). One of the significant wear phenomena identified in these analyses is adhesion wear, in which particles detach from the brake friction material after friction contact with the disk, then reattach. Abrasive wear, on the other hand, is characterized by scratch-like wear caused by hard particles detaching from fractures and relatively softer areas on the surface [33,34]. Figure 8a shows SEM microscopic images of the worn surfaces of BPLY8, Figure 8b BPLY12, Figure 8c BPLY16, and Figure 8d BPLY20 samples. The areas enclosed within the red lines in these images represent the contact surfaces that occur between the brake pad and brake disk during braking, which we can call the primary plateau. The larger these contact surfaces, the higher the friction coefficient, and the more stable it becomes. For the samples Figure 8b-d shown in Figure 8 (excluding BPLY8), significantly larger contact surface areas were observed at different fiber ratios. This situation, as shown in Figure 7, may be due to the loose fiber-matrix bonding resulting from the insufficient fiber ratio in the BPLY8 sample.

The separation and disintegration of plateaus, which we can call secondary plateaus, located outside the contact surface, may also be due to the lower strength of the material beneath the contact surface. If this occurs to a severe degree, partial bending of the friction layer may occur, along with disintegration [35]. The abrasive wear mechanism can cause microcracks and deformations on the brake friction material or disk surface. These deformations are a significant factor in determining the lifespan of brake friction materials or brake disks [36]. One of the significant factors in the emergence of abrasive wear is the effect on the composite material’s bonding performance, due to aggregation resulting from the increased filler ratio. Abrasive wear, characterized by surface material flaking, can cause deep grooving. This mechanism can lead to increased friction resistance and wear severity in brake friction materials [37]. In the developed brake friction materials, a partial abrasive wear trace was observed in the BPLY20 sample shown in Figure 8d. However, this is not severe. The evidence that it is not severe is that the wear rate values are the lowest for this sample.

In Figure 8, the local deep pits within the orange quadrilateral region in images Figure 8a–c are structural deformation areas that appeared on the friction surface after the experiment. Deep pits are a mechanism that can partially occur on the surface areas of brake friction materials during braking. The emergence of this mechanism can be described as the separation of different types of components within the matrix due to mechanical or temperature increase effects, causing them to be released into the external environment.

## 4. Conclusions

Four different bio composite brake pad samples, based on loofah fiber, a renewable biomass, were developed: BPLY8, BPLY12, BPLY16, and BPLY20. The performance criteria of these developed bio composite vehicle brake pads, such as friction coefficient and wear rate, were compared. In addition, macroscopic and microscopic SEM analyses were performed on hardness, disk roughness during braking, and wear surfaces. The following results were obtained from these experiments and tests:

1. All samples yielded stable and acceptable friction coefficient values within the industrial standard range. The maximum friction coefficient was observed in the BPLY8 sample, while the other samples showed lower, more stable friction coefficients.
2. The wear rate values for all samples were within industrial limits. The wear rate decreased with increasing fiber content in the samples. It can be said that the increase in the proportion of fibers and the resulting increase in reinforcing properties, as well as the increase in sample hardness, are particularly effective in reducing the wear rate.
3. When the disk roughness differences were evaluated, as the fiber reinforcement in the sample increased, the differences in Ra and Rz roughness increased, exhibiting a difference within reasonable limits. The increase in roughness values may be due to the partial increase in sample hardness.
4. In the macroscopic analysis of the worn surface of the developed brake pad, a crack was detected along the entire length of the BPLY8 sample due to the insufficient reinforcing property caused by the low fiber ratio resulting from the mechanical loads generated during braking. It was concluded that the use of 8% squash fiber is not suitable in this design.
5. In SEM analyses, it was determined that more integral and wider friction contact surfaces appeared in the samples other than the BPLY8 sample. Partial fiber shrinkage occurred in some of the samples, and a shallow abrasive wear trace was observed in the BPLY20 sample.

## Figures and Tables

**Table 1:** Biopolymer brake pad composite material mixing ratios (wt%).

		Sample Code			
Function	Material	BPLY8	BPLY12	BPLY16	BPLY20
Fiber	Loofah fiber	8	12	16	20
Filler	Calcite	21	19	17	15
	Barite	21	19	17	15
Binder	Resin	20	20	20	20
Lubricant	Graphite	4	4	4	4
Friction modifier	Alumina	10	10	10	10
	Brass Powder	6	6	6	6
	Bronze Powder	6	6	6	6
	Cashew	4	4	4	4

## Data Availability

The data presented in this study are available on request from the corresponding author.
